# Effects of the Rainfall Intensity and Slope Gradient on Soil Erosion and Nitrogen Loss on the Sloping Fields of Miyun Reservoir

**DOI:** 10.3390/plants12030423

**Published:** 2023-01-17

**Authors:** Lei Wang, Yan Li, Jiajun Wu, Zhizhuang An, Linna Suo, Jianli Ding, Shuo Li, Dan Wei, Liang Jin

**Affiliations:** 1 Institute of Plant Nutrition, Resources and Environment, Beijing Academy of Agricultural and Forestry Sciences, Beijing 100097, China; 2 College of Resources and Environmental Sciences, Agricultural University of Hebei, Baoding 071000, China

**Keywords:** rainfall intensity, slope gradient, soil erosion, nitrogen loss

## Abstract

Environmental loss is primarily caused by soil, water, and nutrient loss, and runoff is associated with nutrient transport and sediment loss. Most existing studies have focused on one influencing factor, namely slope gradient or rainfall intensity, for slope erosion and nutrient loss, but the joint effects of the two factors have rarely been researched. In this context, the impact of slope gradients (0°, 5°, 10°, and 15°) and rainfall intensities (30, 40, 50, 60, 70, and 80 mm/h) on soil erosion and nutrient loss on the sloping fields of Miyun Reservoir were explored using the indoor artificial rainfall simulation testing system. Based on the results of the study, the variation of runoff coefficient with slope gradient was not noticeable for rainfall intensities <40 mm/h; however, for rainfall intensities >40 mm/h, the increased range of runoff coefficient doubled, and the increase was the fastest under 0° among the four slope gradients. The slope surface runoff depth and runoff rate showed positive correlations with the rainfall intensity (r = 0.875, *p* < 0.01) and a negative correlation with the slope gradient. In addition, the cumulative sediment yield was positively related to the slope gradient and rainfall intensity (r > 0.464, *p* < 0.05). Moreover, the slope surface runoff-associated and sediment-associated loss rates of total nitrogen (TN) rose as the rainfall intensity or slope gradient increased, and significant linear positive correlations were found between the runoff-associated TN loss rate (NLr) and the runoff intensity and between the sediment-associated NLr and the erosion intensity. In addition, there were positive linear correlations between slope runoff-associated or sediment-associated TN loss volumes and rainfall intensity, surface runoff, and sediment loss volumes, which were highly remarkable. The slope gradient had a significant positive correlation with the slope surface runoff-associated TN loss at 0.05 (r = 0.452) and a significant positive correlation with the sediment-associated TN loss at the level of 0.01 (r = 0.591). The rainfall intensity exhibited extremely positive correlations with the slope surface runoff-associated and sediment-associated TN loss at 0.01 (r = 0.717 and 0.629) Slope gradients have less effect on nitrogen loss on sloped fields than rainfall intensity, mainly because rainfall intensity affects runoff depth. Based on the findings of this study, Miyun Reservoir may be able to improve nitrogen loss prevention and control.

## 1. Introduction

Soil erosion is a significant environmental issue worldwide and a major cause of ecosystem degradation [1]. In recent years, the soil erosion mechanism and shallow water hydrodynamics have been widely researched through experiments. According to the estimation [2], the global average soil erosion in 2012 was about 3.59 × 10^10^ t. The soil erosion rate (Er) on sloped fields, especially in agricultural areas, is the highest [3]. Erosion of soil on sloping fields can negatively affect soil utilization and agricultural production, resulting in reduced soil fertility and productivity and agricultural non-point source pollution.

Currently, a wide range of studies on soil erosion has been conducted by scholars [4,5,6] through indoor tests, field monitoring, and numerical simulation and analysis. In the case of soil erosion, Koiter et al. [7] investigated the effect of soil surface properties on particle size and carbon and found that fine particles and carbon are more abundant in eroded sediment than in the original soil. It has been demonstrated by Ding and Huang [8] that slope roughness influences soil erosion processes as well as particle size distribution. The evaluation and simulation of soil erosion require the consideration of both cumulative sediment yield and particle size. Zhang et al. [9] employed five rare earth elements as tracers to simulate sediment dynamics under rainfall conditions and found that the runoff transport process controls soil erosion. The results of the above studies indicate that sediment separation, transport, settlement, and re-separation occur simultaneously, and these processes are always in a dynamic state. Generally, sediment is transported by rolling or suspension in areas subject to surface erosion. The intensity of rainfall and the slope gradient are the two major factors controlling the hydrological process. Many studies have shown that Er increases with an increase in slope gradient. As shown in the early universal soil loss equation (USLE) and the revised one [10], there is a power-function linear relationship between soil erosion and the slope gradient. FOX and Bryan [11] researched the variation trend of the soil Er with the slope gradient and monitored the change of the runoff velocity in sandy loam soil. It was found that soil loss is related to runoff velocity, and runoff migration capacity limits soil Er. Mahmoodabadi and Sajjadi [12] studied the effects of rainfall intensity, slope gradient, and particle size distribution on the contribution of splash and scouring. The study revealed that rainfall erosion has a transport capacity limitation on gentle slopes, while separation capacity is a limitation on steep slopes. Several factors interact to determine the threshold of sediment transport capacity, including runoff depth, runoff velocity, sediment concentration, sediment particle size, and settling velocity. The threshold of transportation capacity of runoff and sediment has not been determined by traditional research on runoff and sediment. Nevertheless, quantitative estimations of the runoff and sediment yields of different river basins are extremely important.

Rainfall intensity and slope gradient affect runoff and sediment, ultimately leading to soil nutrient loss. Most studies have shown a positive correlation between rainfall intensity and nutrient loss [13,14,15]. There are two main ways in which soil nutrients are lost. At low rainfall intensities, soluble nutrients migrate with runoff, while at high rainfall intensities, soil nutrients migrate with runoff in sediment form [16]. Soil nutrient loss rises with the increase in the slope gradient, but when the slope gradient reaches the threshold value, nutrient loss declines with the rise of the slope gradient, indicating that there is a critical slope gradient for nutrient loss [17]. However, it has been noted that the loss of different nutrients in soil exhibits different characteristics from the loss of runoff and sediment. Wu et al. [18] revealed in their study that during rainfall, the main pathway for nitrogen loss is runoff, while that of phosphorus is sediment. On the contrary, Wang et al. and Wu et al. [19,20] held that surface runoff is the main pathway for soil phosphorus loss in loess-sloping fields. Most of the above studies mainly concentrate on one influencing factor, the slope gradient or rainfall intensity, for slope erosion and nutrient loss, but the combined effects of the two influencing factors have rarely been researched.

Miyun Reservoir is a major surface drinking water source for Beijing, ensuring the safety of the capital’s water sources. Based on statistics, soil erosion has affected an area of 309.97 km^2^ in the Miyun Reservoir, which accounts for 20.9% of the total area of Miyun District. Miyun Reservoir shows a high soil erosion intensity and a high risk of non-point source pollution, but little research has been conducted to explore soil erosion on the sloping fields of Miyun Reservoir at present. Based on the assumption that slope and rainfall intensities increase, runoff and sediment yield increase, nitrogen loss increases, and runoff is the main mechanism for nitrogen loss, we assume that nitrogen loss increases as slope and rainfall intensities increase. This study aims to test the joint effects of slope gradient and rainfall intensity on the soil of the sloping field at Miyun Reservoir. The slope surface runoff, sediment yield, and nitrogen loss were monitored during rainfall to generate the relevant dynamic curves, and the differences between surface runoff-associated nitrogen loss and sediment-associated nitrogen loss were compared. In addition, the slope gradient and rainfall intensity impact mechanisms on the variation law of nitrogen loss were examined in detail. This study is expected to provide supportive data and a scientific basis for the regional maintenance of soil quality and control over water eutrophication.

## 2. Materials and Methods

### 2.1. Experiment Facility and Materials

**Figure 1:**
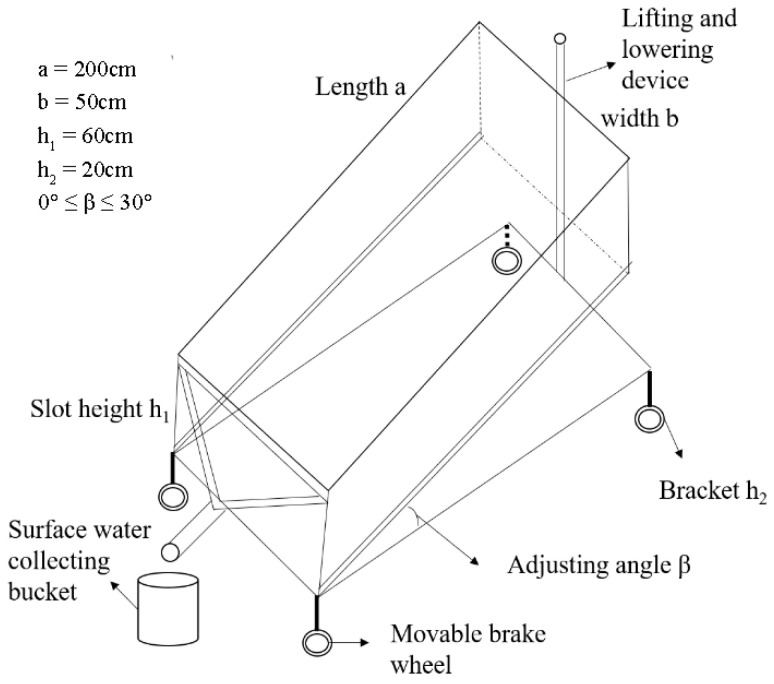
Schematic diagram of a runoff slot.

In this research, an experiment was performed from May to September 2022 at the simulation test base of the Beijing Academy of Agricultural and Forestry Sciences in Haidian District, Beijing, China. The rainfall equipment was the QYJY-502 portable automatic artificial simulation rainfall system (mainly composed of rainfall nozzles, water supply pipelines, pressure gauges, return valves, water supply pumps, stainless steel brackets, on–off valves, etc.) developed by Xi’an Qingyuan Measurement and Control Technology Co., Ltd., with a rainfall height of 5 m, a rainfall uniformity coefficient of over 80%, and a continuous variation range of rainfall intensity of 15–120 mm/h. Runoff plots (length × width × height = 200 cm × 50 cm × 60 cm) (Figure 1), three-dimensional simulated soil plots composed of welded steel plates, were automatically designed by QYJY-502 according to its effective rainfall area with a variable slope that could be flexibly adjusted in the range of 0–30°. The runoff slots were placed in parallel and covered with the same rainfall intensity for repeated calculation. After that, slope runoff and sediment were collected through the triangular outlet at the top of the runoff slots.

### 2.2. Soil Sampling

Soil samples were collected from Taishizhuang Village Non-point Source Pollution Prevention and Control Base (117°6′42.08″ E, 40°32′22.02″ N) around Miyun Reservoir, Miyun District, Beijing, China. The river in the north is the Chao River, and the river in the south is the Qingshui River. The climate type belongs to the warm temperate semi-humid monsoon climate. The average annual temperature of multiple years was 9–10.5 °C. The frost-free period was 180 days. The average annual rainfall of multiple years was about 624 mm and was mostly concentrated from June to September. The rainfall in the three months of the flood season accounted for ~65–75% of the annual rainfall. In the study area, sandy loam soil is the dominant soil type, with its basic physicochemical properties being as follows: pH = 6.33, SOM content = 9.97 g/kg, STN content = 0.448 g/kg, rapidly available phosphorous content = 4.55 mg/kg, and rapidly available potassium content = 45.9 mg/kg, The soil particles are mainly sand particles (about 76.57%), followed by silt particles (about 16.46%), and clay particles (about 6.97%) [21].

The undisturbed soil relocation method was employed to move the test soil into the runoff slot, and 10 layers (50 cm thick) of soil were collected from the surface to the deeper soil layer at 5 cm intervals and bagged. The bulk density was then determined using the cutting-ring method. The runoff slot was filled with the corresponding soil layer in the subsequent step to ensure a consistent bulk density. Before the rainfall test, the runoff slot was left standing for a period to allow the soil to sink naturally and regain its natural characteristics. After that, soil samples were collected, and the soil moisture content was determined to ensure the same initial moisture content of the test soil before each rainfall test.

### 2.3. Experimental Design

In this experiment, six rainfall intensity treatments of 30, 40, 50, 60, 70, and 80 mm/h were designed, with four slope gradients of 0°, 5°, 10°, and 15°, and each treatment group was set up accordingly. The soil runoff slot was covered with a tarpaulin prior to each rainfall test, and the rainfall intensity was then calibrated. Upon reaching the target rain intensity and uniformity (>90%), the tarpaulin was removed while the timing began, and a stopwatch recorded the slope runoff and sediment generation time. Slope runoff and sediment samples were collected every 5 min, and the collection took 60 min in total. In addition, the runoff volume was measured. Next, the collected runoff and sediment samples were settled for at least 24 h, after which the supernatant was poured out from the container, and the remaining wet sediment was dried in an oven at 65 °C and weighed.

### 2.4. Experimental Method

After the simulated rainfall, the runoff volume was measured, and then the runoff bucket was thoroughly stirred, from which 500 mL of the water–sediment mixed sample was put into a plastic bottle. The supernatant from the precipitated samples was placed in a plastic bottle in a refrigerator at 4 °C for chemical analysis. Following air drying, the remaining sediment was sampled and placed into a sealed bag, which was also stored in the refrigerator for chemical analysis. Later, the sediment in the 500 mL sample was separated from the water, dried in the oven, and weighed. Finally, nutrient content in runoff and sediment was determined using conventional methods, total nitrogen (TN) in runoff was detected by potassium persulfate oxidation–ultraviolet spectrophotometry, and TN in sediment was examined by semi-micro Kjeldahl assay.

### 2.5. Statistical Analysis

The data were recorded and processed using Excel, and comparisons of the groups were performed by a one-way analysis of variance (one-way ANOVA) with a Duncan multiple variable test (*p* < 0.05). The correlation coefficients were calculated by the SPSS 26 software (SPSS Inc., Chicago, IL, USA) through Pearson’s correlation and a two-tailed *t* test (*p* < 0.05 and *p* < 0.01). Origin 2021 was used for plotting data.

## 3. Results

### 3.1. Characteristics of Slope Surface Runoff Generation

Under the designed test conditions, the initial slope surface runoff generation time (Ts) showed an obvious regularity (Table 1), i.e., it varied with the slope gradient and rainfall intensity. As illustrated in Table 1, under the same slope gradient, Ts decreased with the increase in the rain intensity (30 > 40 > 50 > 60 > 70 > 80 mm/h in descending order). With the slope gradient of 5° as an example, Ts declined by 15.09, 10.74, 9.55, 5.83, and 0.71 min with increased rainfall intensity. Similar trends were observed under other slope gradients, but the decreasing degree gradually diminished with an increase in slope gradient. Under the same rainfall intensity, the overall Ts decreased with an increase in slope gradient (0° > 5° > 10° > 15° in descending order). Except for rainfall intensity of 40 mm/h, Ts decreased gradually with an increase in rainfall intensities. A close relationship between Ts and rainfall intensity was attributed to the presence of different runoff generation modes on the slope surface. Runoff was generated through two distinct mechanisms: saturation excess under a low rainfall intensity and infiltration excess under a high rainfall intensity. As the slope gradient rose, the horizontal component of runoff gravity along the slope direction increased, which could accelerate the runoff velocity to advance runoff generation.

**Figure 2:**
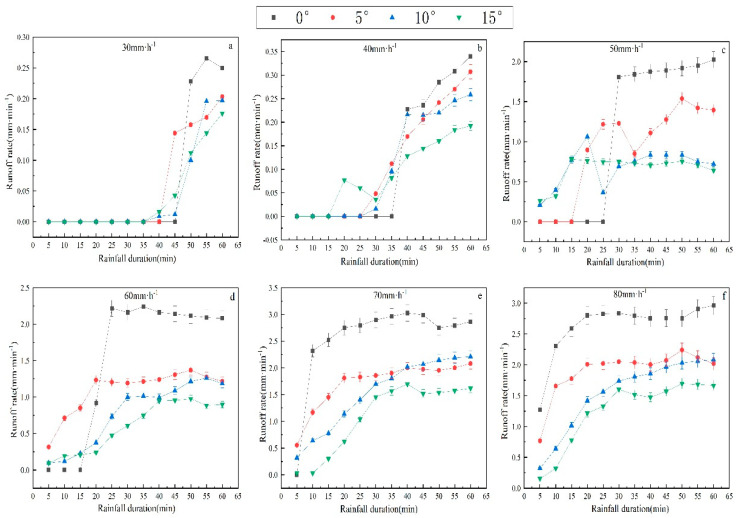
Runoff rate as a function of time under different treatments (slope gradient and rainfall intensity). The error bars refer to the standard deviation. Runoff rate as a function of time under 30 mm·h^−1^ (**a**). Runoff rate as a function of time under 40 mm·h^−1^ (**b**). Runoff rate as a function of time under 50 mm·h^−1^ (**c**). Runoff rate as a function of time under 60 mm·h^−1^ (**d**). Runoff rate as a function of time under 70 mm·h^−1^ (**e**). Runoff rate as a function of time under 70 mm·h^−1^ (**f**).

The surface runoff rate (Rr) was increased with the extension of rainfall duration and finally stayed relatively stable in a dynamic range (Figure 2). The higher the rainfall intensity, the shorter the time it took for Rr to reach a stable range. The initial Rr was very low, as the soil sealing layer had not formed at the beginning of the rainfall. At this initial infiltration-excess stage, the runoff infiltration rate (Ir) was high, and soil erosion was mainly caused by raindrop splash. The Ir was higher than those at other stages, but the Rr was lower. With soil surface sealing and the generation of slope surface runoff, soil erosion was mainly manifested as surface erosion with gradual development. Then the Ir dropped down rapidly, and Rr was elevated correspondingly.

The depth of slope surface runoff rose with the increase in the rainfall intensity but decreased with the increase in the slope gradient. It may be that under the same slope gradient, the rain-receiving area of runoff slots is the same, whereas, under the same rainfall intensity, the rain-receiving area of runoff slots reduces with the increase in slope gradient, resulting in a reduction in Rr. As shown in Table 2, the average Rr was elevated with the rise in the rain intensity, and the rain intensity exerted a more significant influence on the Rr than the slope gradient. Under the same rain intensity but different slope gradients, the Rr difference was substantial, but when the rain intensity was less than 50 mm/h, there was a relatively small Rr difference between different slope gradients.

**Figure 3:**
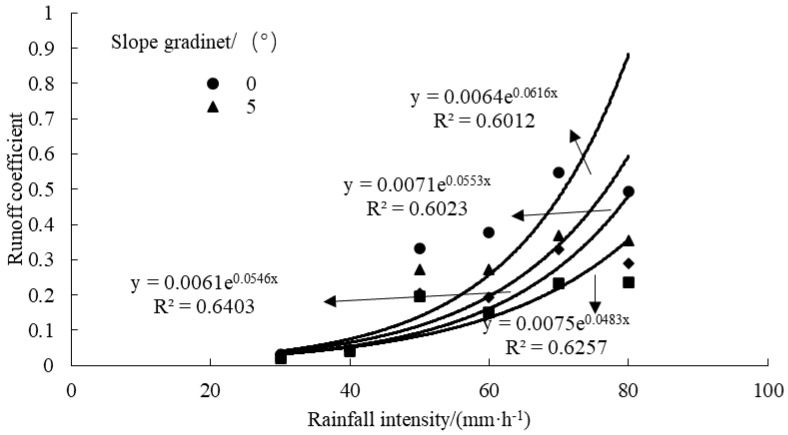
Dynamic characteristic curve of the runoff coefficient changing with the rainfall intensity and slope gradient.

Based on the data in Table 1, the runoff coefficient (cumulative runoff/cumulative rainfall) was calculated, and the dynamic characteristic curve of the runoff coefficient changing with the rainfall intensity and slope gradient was plotted (Figure 3). As revealed by Figure 3, the variation of the runoff coefficient showed an exponential correlation with the rainfall intensity, displaying a correlation coefficient of >0.78, and as the rainfall intensity became higher, the range of the runoff coefficient was increased. The runoff coefficient was the largest under the slope gradient of 10°, showing the closest correlation. According to the variation law of runoff coefficient under six rainfall intensities, in the case of the rainfall intensity <40 mm/h, the variation of runoff coefficient with the slope gradient was not noticeable, but in the case of the rainfall intensity >40 mm/h, the increased range of the runoff coefficient doubled, and the increase was the fastest under 0° among the four slope gradients.

### 3.2. Characteristics of Sediment Loss

**Figure 4:**
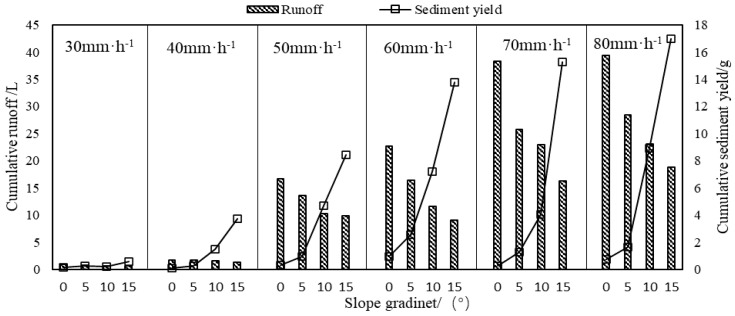
Cumulative runoff and sediment yield under different treatment conditions.

As shown in Figure 4, cumulative sediment yields under different treatments revealed that the cumulative sediment yield was positively correlated with the slope gradient and rainfall intensity (*r* > 0.464, *p* < 0.05), and its increase rate was high under the high rainfall intensity. Hydraulic erosion is the major contributor to eroded sediment yield on the sloping fields, displaying a positive correlation between them. In addition, the cumulative runoff depth was positively related to the rainfall intensity (*r* = 0.875, *p* < 0.01), but under the same rainfall intensity, the cumulative runoff depth declined with the increase in the slope gradient, suggesting that the slope gradient is a crucial influencing factor. Sediment transport capacity is an essential indicator of hydraulic erosion. The eroded sediment concentration (Sc) in the unit runoff depth was calculated to analyze the internal relationship between the runoff depth and soil erosion (Table 3). Under the same rainfall intensity, the average Sc displayed a remarkable positive correlation with the slope gradient (*r* = 0.697, *p* < 0.01). On steep slopes, the average Sc rises as the rainfall intensity increases and the value peaks under the rainfall intensity of 80 mm/h. However, the maximum value of average Sc on gentle slopes appears under the rainfall intensity of 60 mm/h, i.e., the ultimate value appears before the highest rainfall intensity, implying that higher sediment transport capacity cannot always lead to higher runoff depths and sediment yields. As the slope gradient further rises, the rain-receiving area of runoff slots is reduced, the Ir is up-regulated, and the Rr is down-regulated. In this study, the higher soil Er was associated with a higher sediment transport capacity, suggesting that the slope gradient determines the relationship between rainfall intensity and average Sc on gentle slopes.

**Figure 5:**
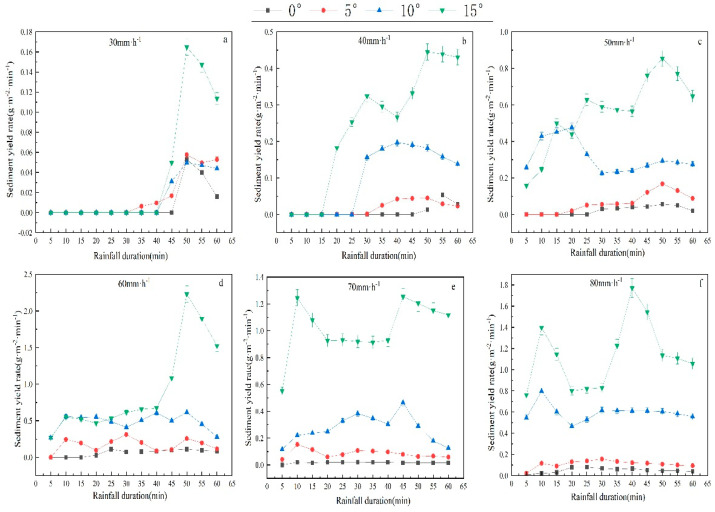
Sediment yield rate as a function of time under different treatments (slope gradient and rainfall intensity). The error bars refer to the standard deviation. Sediment yield rate as a function of time under 30 mm·h^−1^ (**a**). Sediment yield rate as a function of time under 40 mm·h^−1^ (**b**). Sediment yield rate as a function of time under 50 mm·h^−1^ (**c**). Sediment yield rate as a function of time under 60 mm·h^−1^ (**d**). Sediment yield rate as a function of time under 70 mm·h^−1^ (**e**). Sediment yield rate as a function of time under 70 mm·h^−1^ (**f**).

As illustrated in Figure 5, the Er generally displayed an increasing and then decreasing trend with the extension of rainfall duration. At the initial runoff infiltration stage, the slope surface runoff depth was small, but soil erosion appeared in runoff slots. After the runoff–sediment mixed samples were collected, sediment migrated from the upper part of the slope to the bottom. When runoff and sediment flew out of the runoff slots, the Er at the early stage would be higher. As the rainfall test progressed, runoff infiltration became saturated, Rr was constant, and the erosion intensity of raindrops and slope runoff on slope soil was relatively stable. Once denudation reached the limit, all the fine soil particles on the slope surface were transported, which decreased sediment content and Er in the subsequent slope surface runoff as rainfall duration extended. During the test, some small-scale blocks collapsed, inducing the fluctuation of Er.

### 3.3. Characteristics of Slope Surface Runoff-Associated Nitrogen Loss

**Figure 6:**
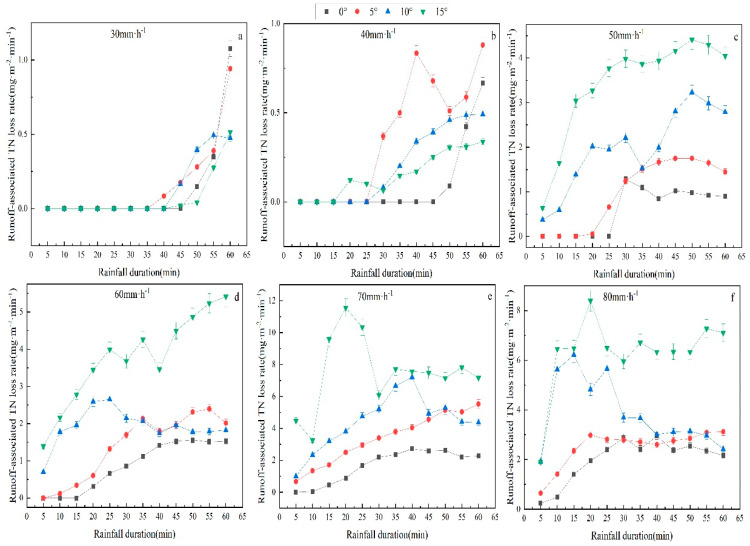
Runoff-associated TN loss rate as a function of time under different treatments (slope gradient and rainfall intensity). The error bars refer to the standard deviation. Runoff-associated TN loss rate as a function of time under 30 mm·h^−1^ (**a**). Runoff-associated TN loss rate as a function of time under 40 mm·h^−1^ (**b**). Runoff-associated TN loss rate as a function of time under 50 mm·h^−1^ (**c**). Runoff-associated TN loss rate as a function of time under 60 mm·h^−1^ (**d**). Runoff-associated TN loss rate as a function of time under 70 mm·h^−1^ (**e**). Runoff-associated TN loss rate as a function of time under 70 mm·h^−1^ (**f**).

As revealed by Figure 6, the variation characteristics of TN loss rate (NLr) with rainfall duration under different rainfall intensities and slope gradients manifested that, under the same rainfall intensity, the NLr displayed very similar dynamic variation laws. At the early stage of rainfall, the NLr showed a stepwise uptrend, and the higher the rainfall intensity, the more drastic the increase, and then the NLr began to decline gradually with time and remained stable. The comparison of the NLr under the four rainfall intensities (50, 60, 70, and 80 mm/h) denoted that the NLr under the rainfall intensity of 80 mm/h reached the maximum value, and the NLr under the rainfall intensity of 80 mm/h at each time point was higher than that under the other three rainfall intensities, indicating that the heavy rainfall indeed causes the severe loss of soil nutrients. By comparison, it was discovered that the variation curves of the NLr under the rainfall intensities of 30 mm/h and 40 mm/h showed no noticeable difference, which may be due to the small effect of the low rainfall intensity on soil nutrient loss rate.

### 3.4. Characteristics of Sediment-Associated Nitrogen Loss

**Figure 7:**
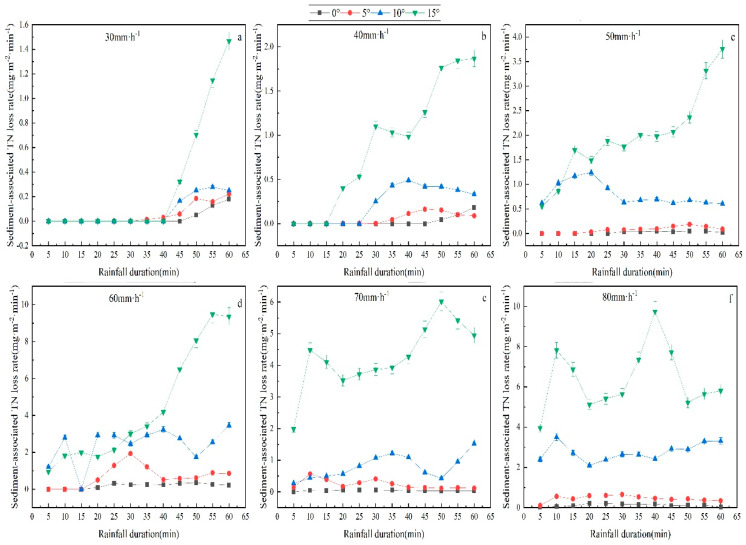
Sediment-associated TN loss rate as a function of time under different treatments (slope gradient and rainfall intensity). The error bars refer to the standard deviation. Sediment -associated TN loss rate as a function of time under 30 mm·h^−1^ (**a**). Sediment -associated TN loss rate as a function of time under 40 mm·h^−1^ (**b**). Sediment -associated TN loss rate as a function of time under 50 mm·h^−1^ (**c**). Sediment -associated TN loss rate as a function of time under 60 mm·h^−1^ (**d**). Sediment -associated TN loss rate as a function of time under 70 mm·h^−1^ (**e**). Sediment -associated TN loss rate as a function of time under 70 mm·h^−1^ (**f**).

In the rainfall process, nitrogen in the soil will be partially lost in the runoff but lost mainly in sediment. The sediment carried by runoff could potentially become a source of nitrogen in nearby rivers or lakes, which poses a severe threat to the water environment. It is, therefore, necessary to investigate the dynamic variation law of nitrogen in sediment under rainfall conditions. This study analyzed the NLr under different rainfall intensities and slope gradients (Figure 7). The results revealed that under the rainfall intensities of 30, 40, and 50 mm/h, the NLr gradually rose as the rainfall duration extended, while under the rainfall intensities of 60, 70, and 80 mm/h, the NLr first rose and then tended to be stable, suggesting that the NLr is elevated with the increase in the rainfall intensity, i.e., a positive correlation. In the meantime, the greater the slope gradient, the higher the NLr. To summarize, the NLr rises with increased rainfall intensity and slope gradient.

### 3.5. Correlations of NLr with Runoff Intensity and Sediment Yield

**Figure 8:**
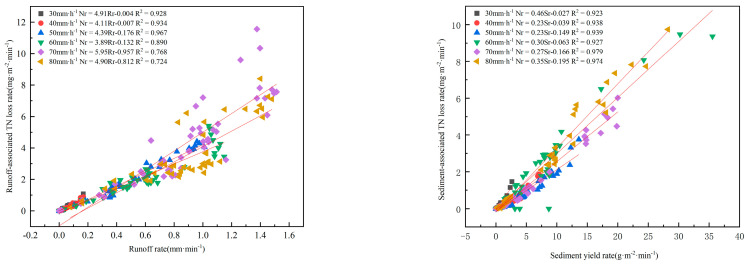
Correlations of NLr with runoff intensity and Sr.

In order to determine the relationship between slope surface TN loss associated with runoff and sediment, regression analyses were conducted using the nitrogen loss rate in runoff and sediment as well as the runoff intensity and erosion intensity, respectively. After comparison, the equation with the highest correlation coefficient was selected (Figure 8). The intensity of rainfall and slope gradient affects runoff and erosion intensity, thereby influencing the NLr to some extent. As revealed by the results of the regression analyses, linear correlations could be detected between the runoff-associated TN loss and the runoff intensity and between the sediment-associated NLr and the erosion intensity, respectively, both of which were positive. Under the rainfall intensities of 30, 40, 50, 60, 70, and 80 mm/h, the relationship between the runoff-associated NLr and the runoff intensity could be represented as follows: NLr = 4.91Rr-0.004 (*r*^2^ = 0.928), NLr = 4.11Rr-0.007 (*r*^2^ = 0.934), NLr = 4.39Rr-0.176 (*r*^2^ = 0.967), NLr = 3.89Rr-0.132 (*r*^2^ = 0.890), NLr = 5.95Rr-0.957 (*r*^2^ = 0.768), and NLr = 4.90Rr-0.812 (*r*^2^ = 0.724). When the rainfall intensity rose from 30 mm/h to 80 mm/h, the correlation coefficient of the correlation equation between runoff-associated NLr and the runoff intensity was reduced, suggesting that the variation range of runoff-associated NLr declines with the increase in the runoff intensity, and the influence of the rainfall intensity on runoff-associated NLr weakens but remains extremely positively significant. Therefore, it is evident that runoff substantially impacts TN loss.

Under the rainfall intensities of 30, 40, 50, 60, 70, and 80 mm/h, the correlation between the sediment-associated NLr and Sr could be presented as follows: NLr = 0.46Sr-0.027 (*r*^2^ = 0.923), NLr = 0.23Sr-0.039 (*r*^2^ = 0.938), NLr = 0.23Sr-0.149 (*r*^2^ = 0.939), NLr = 0.30Sr-0.063 (*r*^2^ = 0.927), NLr = 0.27Sr-0.166 (*r*^2^ = 0.979), and NLr = 0.35Sr-0.195 (*r*^2^ = 0.974). In sediment, the correlation coefficient of the equation between the sediment-associated NLr and the Sr rose with the increase in the Sr. These results indicated that the variation range of sediment-associated NLr is enlarged with the increase in the sediment yield, and the influence of the rainfall intensity on sediment-associated NLr becomes more significantly positive. The higher the rainfall intensity, the more significant difference in Sr between slope gradients of 0° and 15°, suggesting that the slope gradient strengthens its influence on sediment-associated NLr as rainfall intensity increases.

### 3.6. Joint Effects of the Slope Gradient, Rainfall Intensity, Runoff Depth, and Sediment Yield on Surface Runoff-Associated and Sediment-Associated TN Loss

A correlation analysis was conducted using SPSS 20.0 (Table 4) to examine the relationship between slope gradient, rainfall intensity, runoff depth, sediment yield, and slope runoff- and sediment-associated TN loss. It examined the correlations between slope gradient, rainfall intensity, runoff depth, and sediment yield with slope runoff-associated and sediment-associated TN loss and the total loss.

As denoted in Table 4, the slope gradient had a significant positive correlation with the slope surface runoff-associated TN loss at 0.05 (r = 0.452) and a significant positive correlation with the sediment-associated TN loss at the level of 0.01 (r = 0.591). It is evident that the slope gradient on the sloping fields of Miyun Reservoir impacts both slope surface runoff and sediment-associated TN loss, with the latter experiencing a more significant effect. Unlike the slope gradient, the rainfall intensity exhibited extremely positive correlations with the slope surface runoff-associated and sediment-associated TN loss at 0.01 (*r* = 0.717 and 0.629, respectively). It can be seen that the rainfall intensity affects both the slope surface runoff-associated and sediment-associated TN loss, and the effect on the former is more evident. The surface runoff depth and sediment yield were positively related to the slope surface runoff-associated and sediment-associated TN loss at 0.01 (*r* = 0.458 and 0.785, respectively). In summary, slope surface runoff-associated TN loss could be influenced by three factors (extent of the influence: rainfall intensity > surface runoff depth > slope gradient), and sediment-associated TN loss could also be affected by three factors (extent of the influence: sediment yield > rainfall intensity > slope gradient).

Regression analyses on the measured rainfall data on each field were performed by SPSS20.0, and regression models (1) and (2) were obtained:

*S*_1_ = 0.465*G* + 0.320*I −* 0.082*V*_1_
*−* 2.326 (*r* = 0.849, *F* = 17.228) (1)

*S*_2_ = 1.109*G* + 0.455*I* + 0.837*V*_2_
*−* 5.511 (*r* = 0.876, *F* = 21.942) (2)

where *S*_1_ is slope surface runoff-associated TN loss (mg), *S*_2_ is sediment-associated TN loss (mg), *G* is the slope gradient (°), *I* is the rainfall intensity (mm/min), *V*_1_ is slope surface runoff depth (L), and *V*_2_ is subsurface runoff depth (L).

Linear correlation equations could accurately describe the joint effects of the slope gradient, rainfall intensity, runoff depth, and sediment yield on the slope surface runoff-associated and sediment-associated TN loss (*r* = 0.849 and 0.876, respectively). The fitting degree of the model was good, and the fitting degree of the slope surface runoff-associated TN loss model was slightly lower than that of the sediment-associated TN loss model.

## 4. Discussion

On the premise of the same initial soil moisture content, the occurrence time of rainfall runoff is mainly associated with the slope gradient and rainfall intensity, and the slope surface infiltration and the rainfall-receiving area of soil slots also affect the occurrence of runoff [4]. The coarse soil of Miyun Reservoir shows poor water retention ability, leading to low soil moisture content. At the initial stage of rainfall, rainwater can be used to moisten the soil and fill the pores of the soil layer, resulting in the runoff on the slope surface after rainfall. In the present study, the initial runoff generation time is the same as in previous studies [22,23,24,25]. Under low rainfall intensity, the soil shows greater infiltration capacity than water supply capacity, and water infiltration is primarily based on the water supply rate. The larger the slope gradient, the greater the gravity in the vertical direction, and the faster the movement of slope surface runoff and subsurface runoff, the shorter the time required for runoff. Under the same slope gradient, the higher the rainfall intensity, the larger the rainfall depth per unit time in soil slots, and the faster the soil can reach saturation, thus facilitating the occurrence of slope surface runoff. Surface runoff moves along a relatively short path and occurs within a very short period under rainfall intensity of 50–80 mm/h.

The slope gradient and rainfall intensity are major players in runoff and sediment generation [26]. As the slope gradient and rainfall intensity rise, erosion sediment generation becomes more sensitive to conditions, and the pivotal influencing factors for erosion alternate between the slope gradient and rainfall intensity [27,28]. It has been shown that rainfall intensity affects runoff depth and raindrop kinetic energy, which alters sediment yield [29]. Rainfall-caused erosion is mainly affected by the splash of raindrops and runoff scouring. With the increase in the rain intensity, raindrops gain more kinetic energy, thus increasing the splash intensity and giving rise to more loose soil particles on the sloping fields [30,31]. Increasing the scouring capacity of runoff can facilitate the transport of these loose soil particles, so the higher the rain intensity, the higher the Er. Cao et al. [32] found that, in addition to the rainfall intensity, the slope gradient is a critical factor for the control of Er. Soil particles on gentle slopes are not susceptible to activation, while those on steep slopes are prone to activation and transport. With a large interparticle gap, loose soil particles exhibit small interparticle cohesion, so they are easily transported by runoff during rainfall. Influenced by infiltration, the small particles on gentle slopes will fill the pores in the soil and facilitate the formation of crust on the surface, thus improving the anti-erosion performance of the surface and reducing the Er.

Additionally, as the runoff velocity and power are higher on steep slopes, soil particles are more easily transported by runoff, and their quantity becomes larger with the increase in the slope runoff depth. Ali et al. [33] found that the runoff transport capacity limits soil erosion, and such a limitation will be reduced by the increase in the runoff depth and Rr on steep slopes. Nonetheless, slope surface runoff cannot transport all original soil particles but only transport those separated by rainfalls. In this study, the analysis results of the various characteristics of TN loss in runoff demonstrated that the NLr in runoff showed a stepwise uptrend with the extension of the rainfall duration, indicating that TN loss in the soil gradually rises and TN content gradually declines. Nitrogen plays an essential role in the growth of plants, and its loss threatens the growth of plants [34,35,36]. Therefore, most fields, especially sloping farmlands where runoff easily occurs, may be affected by continuous rainfall, which will influence crop yields to some extent. At the early stage of runoff generation, the concentration of TN in sediment is relatively high due to a large number of loose particles on the slope surface. As surface crust emerges, TN concentrations gradually decline and tend to be stable, although this stability does not last. Under the conditions of the test design in this study, the TN concentration showed a stepwise downtrend and tended to be stable in a period from about 15 min after runoff generation to the end of rainfall (60 min). However, in the case of a longer rainfall or the seriously damaged slope crust, evident erosion gullies will appear. It is inevitable for the TN concentration to change, and the specific situation needs further investigation. Eroded sediment is a carrier of TN loss, so there is no doubt that the eroded sediment yield is related to the volume of TN loss to a certain extent. A pronounced linear relationship was detected between soil erosion rate and NLr, which makes it possible to analyze nutrient loss based on the loss of slope soil.

Meanwhile, it has been shown [37,38] that the selective erosion of slope soil particles by runoff mainly induces the variation of the TN concentration during runoff generation. Therefore, the variation process of particle concentration in lost sediment can be predicted using the variation process of TN concentration. In this study, the NLr under the same rainfall intensity but different slope gradients indicated that the nutrient concentration often could not reflect the real situation of nutrient loss in a particular land. In other words, under some conditions, although the nutrient concentration is high in the lost sediment, the amount of nutrients lost is not large. Therefore, compared with the nutrient concentration, the nutrient loss rate is considered a better indicator of the loss of soil nutrients in a particular land.

## 5. Conclusions

(1) The slope surface runoff depth and Rr showed positive correlations with the rainfall intensity (r = 0.875, *p* < 0.01) and a negative correlation with the slope gradient. In addition, the total sediment yield was positively related to the slope gradient and rainfall intensity (r > 0.464, *p* < 0.05). In the case of the rainfall intensity <40 mm/h, the variation of the runoff coefficient with the slope gradient was not obvious, but in the case of the rainfall intensity >40 mm/h, the increased range of the runoff coefficient doubled, and the increase was the fastest under 0° among the four slope gradients. The slope surface runoff-associated and sediment-associated NLr rose as the rainfall intensity, or slope gradient, increased. Significant linear positive correlations were found between the runoff-associated NLr and the runoff intensity and between the sediment-associated NLr and the erosion intensity.

(2) Positive correlations of the slope surface runoff-associated or sediment-associated TN loss volume with the rainfall intensity, surface runoff depth, and sediment loss volume were also observed, which were highly remarkable. In addition, there were linear correlations between the slope gradient and the slope surface runoff-associated TN loss volume and sediment-associated TN loss volume, which were all positive, and the latter correlation was more significant. It can be concluded that, compared with the slope gradient, the rainfall intensity exerts a more significant impact on nitrogen loss on sloping fields, mainly by affecting the runoff depth or sediment yield on the slope surface. The cutoff value of the rainfall intensity existed between 50 mm/h and 80 mm/h. When the rainfall intensity exceeded this cutoff value, the slope surface runoff-associated TN loss volume and its proportion to the total loss volume would be notably elevated, and the larger the slope gradient was, the more pronounced this phenomenon would be. Miyun Reservoir’s management personnel could cultivate cover crops around the slopes of 10° to 15° in order to reduce soil erosion efficiently. In the case of rainfall intensities ranging from 30 to 80 mm/h, soil erosion on slopes should be especially closely monitored when rainfall intensities range from 50 to 80 mm/h. Once the rainfall intensity exceeded this value, the erosive ability of slope surface runoff to surface soil would be greatly enhanced, thus increasing the loss of granular nitrogen. 

## Figures and Tables

**Table 1:** Characteristics of runoff depth and erosion under the simulated rainfall.

Rainfall Intensity/ (mm·h^−1^)	Slope Gradient/ (°)	Starting Time of Runoff Occurrence Ts/min	Runoff/L	Sediment Yield/g	Rainfall Intensity/ (mm·h^−1^)	Slope Gradient/ (°)	Starting Time of Runoff Occurrence Ts/min	Runoff/L	Sediment Yield/g
30	0	49.54	0.93	0.14	60	0	18.08	22.67	0.96
	5	44.86	0.84	0.24		5	9.58	16.43	2.54
	10	39.33	0.64	0.22		10	3.64	11.64	7.23
	15	38.11	0.61	0.59		15	2.58	9.04	13.77
40	0	36.52	1.75	0.12	70	0	9.83	38.35	0.25
	5	29.87	1.69	0.26		5	3.75	25.74	1.27
	10	29.55	1.59	1.50		10	2.25	23.02	4.06
	15	19.83	1.33	3.71		15	1.33	16.26	13.88
50	0	27.24	16.64	0.34	80	0	9.83	39.44	0.75
	5	19.13	13.66	0.94		5	3.75	28.46	1.66
	10	4.32	10.27	4.71		10	2.25	23.13	8.93
	15	3.75	9.87	8.41		15	1.33	18.76	17.00

**Table 2:** Average Rr under different slope gradients and rainfall intensities.

Slope Gradient/ (°)	30 mm·h^−1^	40 mm·h^−1^	50 mm·h^−1^	60 mm·h^−1^	70 mm·h^−1^	80 mm·h^−1^
0	0.065 ± 0.003 aD	0.115 ± 0.011 aD	1.099 ± 0.031 aC	1.540 ± 0.084 aB	2.531 ± 0.181 aA	2.642 ± 0.106 aA
5	0.053 ± 0.007 abC	0.111 ± 0.013 aC	0.939 ± 0.102 aB	1.098 ± 0.136 bB	1.771 ± 0.182 bA	1.886 ± 0.084 bA
10	0.045 ± 0.008 bcC	0.102 ± 0.015 aC	0.705 ± 0.142 bB	0.773 ± 0.074 cB	1.554 ± 0.087 bA	1.583 ± 0.156 cA
15	0.040 ± 0.008 dC	0.089 ± 0.015 aC	0.691 ± 0.142 bB	0.640 ± 0.111 cB	1.116 ± 0.136 cA	1.236 ± 0.095 cA

Unit: mm·min^−1^. Note: Different capital letters at the top of the histogram denote the significant differences in Average Rr in different slope gradients at the same rainfall intensities (*p* < 0.05). Different lowercase letters indicate the significant differences in Average Rr at different rainfall intensities at the same slope gradients (*p* < 0.05).

**Table 3:** Average sediment yield rate (Sr) under different slope gradients and rainfall intensities.

Slope Gradient/ (°)	30 mm·h^−1^	40 mm·h^−1^	50 mm·h^−1^	60 mm·h^−1^	70 mm·h^−1^	80 mm·h^−1^
0	0.009 ± 0.003 bB	0.010 ± 0.005 cB	0.024 ± 0.011 dB	0.064 ± 0.012 cA	0.022 ± 0.013 cB	0.052 ± 0.013 cA
5	0.014 ± 0.006 bD	0.018 ± 0.006 cD	0.064 ± 0.011 cC	0.159 ± 0.022 cA	0.085 ± 0.012 cC	0.115 ± 0.018 cB
10	0.017 ± 0.007 bD	0.107 ± 0.026 bD	0.312 ± 0.013 bC	0.461 ± 0.084 bB	0.289 ± 0.055 bC	0.594 ± 0.061 bA
15	0.042 ± 0.011 aE	0.246 ± 0.022 aD	0.564 ± 0.032 aC	0.920 ± 0.076 aB	1.060 ± 0.091 aA	1.060 ± 0.091aA

Unit: g·m^2^·min^−1^. Note: Different capital letters at the top of the histogram denote the significant differences in Average sediment yield rate in different slope gradients at the same rainfall intensities (*p* < 0.05). Different lowercase letters indicate the significant differences in Average sediment yield rate at different rainfall intensities at the same slope gradients (*p* < 0.05).

**Table 4:** Correlation analyses of slope surface runoff-associated and sediment-associated TN loss with the slope gradient, rainfall intensity, runoff, and sediment yield.

	Slope Surface Runoff-Associated TN Loss	Sediment-Associated TN Loss
Slope	0.452 *	0.591 **
Rainfall intensity	0.717 **	0.629 **
Runoff	0.458 **	-
Sediment yield	-	0.785 **

Note: ** *p* < 0.01, * *p* < 0.05.

## Data Availability

Not applicable.
